# Crystal structure of (3,5-dibromo-2-hy­droxyphenyl){1-[(naphthalen-1-yl)carbonyl]-1*H*-pyrazol-4-yl}methanone

**DOI:** 10.1107/S1600536814018601

**Published:** 2014-08-20

**Authors:** Yoshinobu Ishikawa, Yuya Motohashi

**Affiliations:** a School of Pharmaceutical Sciences, University of Shizuoka, 52-1 Yada, Suruga-ku, Shizuoka 422-8526 , Japan

**Keywords:** crystal structure, diaroyl pyrazole, cyclization, stacking inter­action, C—H⋯O hydrogen bonding

## Abstract

In the title compound, C_21_H_12_Br_2_N_2_O_3_, a 1,4-diaroyl pyrazole derivative, the dihedral angles between the naphthalene ring system and the pyrazole ring, the pyrazole and benzene rings, and the naphthalene ring system and benzene ring are 50.0 (2), 51.1 (2) and 1.34 (16)°, respectively. The phenolic proton forms an intra­molecular O—H⋯O hydrogen bond with the adjacent carbonyl O atom. In the crystal, mol­ecules are linked by C—H⋯O hydrogen bonds, forming inversion dimers. The dimers are linked by C—H⋯Br hydrogen bonds, forming double stranded chains along [01-1]. The chains are linked by π–π inter­actions between the pyrazole rings and between the naphthalene and benzene rings [centroid–centroid distances = 3.592 (4) and 3.632 (4) Å, respectively].

## Related literature   

For the biological activity of related compounds, see: Khan *et al.* (2009 ▶); Tu *et al.* (2013 ▶). For related structures, see: Ishikawa (2014 ▶); Ishikawa & Watanabe (2014*a* ▶,*b* ▶,*c* ▶,*d* ▶).
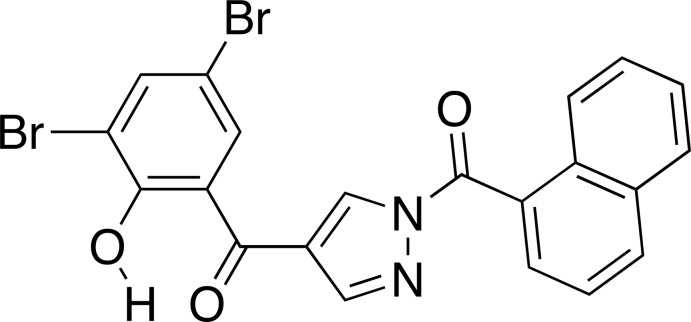


## Experimental   

### Crystal data  

C_21_H_12_Br_2_N_2_O_3_
*M* *_r_* = 500.15
Triclinic, 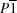

*a* = 7.390 (5) Å
*b* = 8.919 (4) Å
*c* = 14.955 (9) Å
α = 74.61 (4)°
β = 76.71 (5)°
γ = 71.03 (4)°
*V* = 887.5 (9) Å^3^
*Z* = 2
Mo *K*α radiation
μ = 4.61 mm^−1^
*T* = 100 K
0.40 × 0.18 × 0.08 mm

### Data collection  

Rigaku AFC7R diffractometer
Absorption correction: ψ scan (North *et al.*, 1968 ▶) *T*_min_ = 0.448, *T*_max_ = 0.692
5003 measured reflections
4088 independent reflections
3359 reflections with *F* ^2^ > 2.0σ(*F* ^2^)
*R*_int_ = 0.062
3 standard reflections every 150 reflections intensity decay: −0.6%

### Refinement  

*R*[*F* ^2^ > 2σ(*F* ^2^)] = 0.064
*wR*(*F* ^2^) = 0.204
*S* = 1.07
4088 reflections
254 parameters
H-atom parameters constrained
Δρ_max_ = 2.15 e Å^−3^
Δρ_min_ = −2.52 e Å^−3^

### 

Data collection: *WinAFC* (Rigaku, 1999 ▶); cell refinement: *WinAFC*; data reduction: *WinAFC*; program(s) used to solve structure: *SIR92* (Altomare *et al.*, 1994 ▶); program(s) used to refine structure: *SHELXL97* (Sheldrick, 2008 ▶); molecular graphics: *CrystalStructure* (Rigaku, 2010 ▶); software used to prepare material for publication: *CrystalStructure*.

## Supplementary Material

Crystal structure: contains datablock(s) General, I. DOI: 10.1107/S1600536814018601/tk5338sup1.cif

Structure factors: contains datablock(s) I. DOI: 10.1107/S1600536814018601/tk5338Isup2.hkl

Supporting information file. DOI: 10.1107/S1600536814018601/tk5338Isup3.cml

**Fig. 1.**
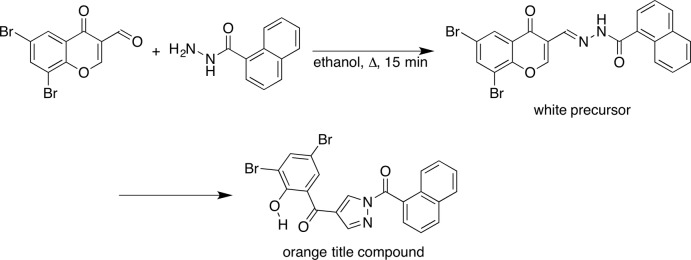
Reaction scheme for the title compound.

. DOI: 10.1107/S1600536814018601/tk5338fig1.tif
Reaction scheme for the title compound.

**Fig. 2.**
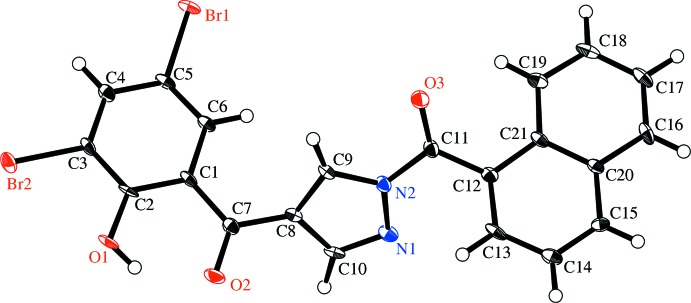
The molecular structure of the title compound, with displacement ellipsoids drawn at the 50% probability level. Hydrogen atoms are shown as small spheres of arbitrary radius.

. DOI: 10.1107/S1600536814018601/tk5338fig2.tif
The mol­ecular structure of the title compound, with displacement ellipsoids drawn at the 50% probability level. Hydrogen atoms are shown as small spheres of arbitrary radius.

**Fig. 3.**
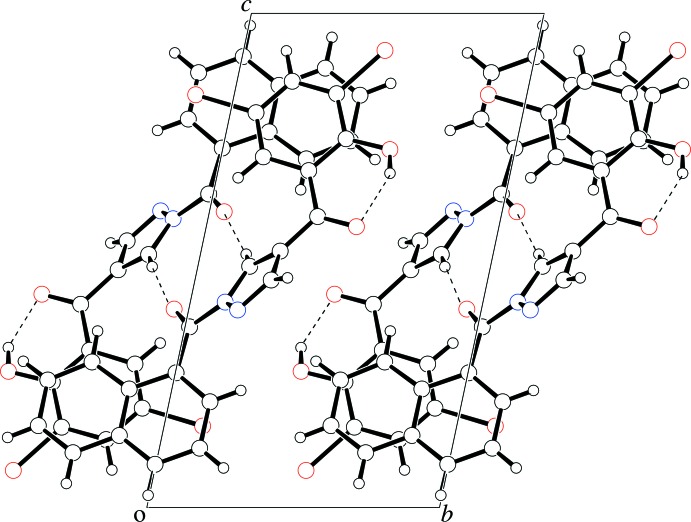
A crystal packing view of the title compound. Intramolecular O—H···O and intermolecular C–H···O hydrogen bonds are represented by dashed lines.

. DOI: 10.1107/S1600536814018601/tk5338fig3.tif
A crystal packing view of the title compound. Intra­molecular O—H⋯O and inter­molecular C–H⋯O hydrogen bonds are represented by dashed lines.

CCDC reference: 1019490

Additional supporting information:  crystallographic information; 3D view; checkCIF report

## Figures and Tables

**Table 1:** Hydrogen-bond geometry (Å, °)

*D*—H⋯*A*	*D*—H	H⋯*A*	*D*⋯*A*	*D*—H⋯*A*
O1—H3⋯O2	0.84	1.85	2.565 (7)	142
C9—H4⋯O3^i^	0.95	2.30	3.227 (8)	165
C16—H9⋯Br2^ii^	0.95	2.88	3.613 (7)	135

Symmetry codes: (i) 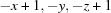; (ii) 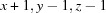.

## supplementary crystallographic information

### S1. Structural commentary

Schiff base derivatives of 3-formyl­chromones have attracted much attention due to their biological functions such as enzyme inhibition (Khan *et al.* 2009; Tu *et al.* 2013). We have recently reported the crystal structures of such compounds (Ishikawa & Watanabe, 2014*a*,*b*,*c*,*d*), which were prepared from condensation reaction of 3-formyl­chromones with aryl­hydrazides.

The reaction of 6,8-di­bromo-3-formyl­chromone (Ishikawa, 2014) with 1-naphtho­ylhydrazide in ethanol gave white solids, and orange crystals were obtained from an aceto­nitrile/ethanol solution of the white solids (Fig. 1). The crystallographic analysis revealed that the structure of the orange crystals is a 1,4-diaroyl pyrazole, as shown in Fig. 2, which should be thermodynamically more stable than that of the white solids. The dihedral angles between the naphthalene and pyrazole rings, the pyrazole and benzene rings and the naphthalene and benzene rings are 50.0 (2), 51.1 (2) and 1.34 (16)°, respectively. The phenolic proton forms an intra­molecular O—H···O hydrogen bond with the adjacent carbonyl O2 atom. In the crystal, the molecules are linked through stacking inter­actions between the pyrazole rings and the naphthalene and benzene rings [centroid–centroid distances = 3.553 (4) and 3.632 (4) Å, respectively, i: –*x* + 2, –*y*, –*z* + 1], and are further connected through inter­molecular C–H···O hydrogen bonds, as shown in Fig. 3. A significant short contact around the bromine atoms is not observed.

The driving force of the intra­molecular cyclization (Fig. 1) should be a resonance energy gain, resulting from the extension of the conjugated system across the entire molecule. The intra­molecular cyclization is not observed for the chromone derivatives with electron-donating substituents (Ishikawa & Watanabe, 2014*a*,*b*,*c*,*d*), and thus the activation energy for the chromone derivative with the electron-withdrawing substituents should be lower than that for ones with electron-donating substituents.

### S2. Synthesis and crystallization

Preparation of the white precursor, (*E*)-*N*'-((6,8-di­bromo-4-oxo-4*H*-chromen-3-yl)methyl­ene)-1-naphtho­hydrazide, is as follows: 1-naphtho­hydrazide (1.1 mmol) and 6,8-di­bromo-3-formyl­chromone (1.1 mmol) were dissolved in 50 ml of ethanol, and the mixture was refluxed with Dean-Stark apparatus for 15 min with stirring. After cooling, the white precipitates were collected, washed with *n*-hexane and dried *in vacuo* (yield 25%). ^1^H NMR (400 MHz, DMSO-*d*_6_): *δ* = 7.60–7.64 (m, 4H), 7.77 (d, 1H, *J* = 6.8 Hz), 8.02–8.05 (m, 1H), 8.11 (d, 1H, *J* = 8.3 Hz), 8.17 (d, 1H, *J* = 2.4 Hz), 8.21–8.23 (m, 1H), 8.45 (d, 1H, *J* = 1.9 Hz), 8.47 (s, 1H), 12.16 (s, 1H). DART-MS (negative mode) calcd for [C_21_H_12_Br_2_N_2_O_3_]: 499.919, found 498.920. The orange crystals suitable for X-ray diffraction were obtained by slow evaporation of an aceto­nitrile/ethanol solution of the white precursor at room temperature.

### S3. Refinement

The O- and C(*sp*^2^)-bound hydrogen atoms were placed in their geometric positions [O—H = 0.84 Å and C—H 0.95 Å, *U*_iso_(H) = 1.2*U*_eq_(O,C)], and refined using a riding model. Three reflections, *i.e*. (4 6 7), (4 6 3) and (4 6 5) were omitted owing to poor agreement. The maximum and minimum residual electron density peaks of 2.15 and 2.52 eÅ^-3^, respectively, were located 0.88 and 1.05 Å from the Br1 and Br2 atoms, respectively.

### Crystal data

**Table:**

C_21_H_12_Br_2_N_2_O_3_	*Z* = 2
*M**_r_* = 500.15	*F*(000) = 492.00
Triclinic, *P*1	*D*_x_ = 1.871 Mg m^−^^3^
Hall symbol: -P 1	Mo *K*α radiation, λ = 0.71069 Å
*a* = 7.390 (5) Å	Cell parameters from 25 reflections
*b* = 8.919 (4) Å	θ = 15.1–17.5°
*c* = 14.955 (9) Å	µ = 4.61 mm^−^^1^
α = 74.61 (4)°	*T* = 100 K
β = 76.71 (5)°	Plate, orange
γ = 71.03 (4)°	0.40 × 0.18 × 0.08 mm
*V* = 887.5 (9) Å^3^	

### Data collection

**Table:**

Rigaku AFC7R diffractometer	*R*_int_ = 0.062
ω–2θ scans	θ_max_ = 27.5°
Absorption correction: ψ scan (North *et al.*, 1968)	*h* = −5→9
*T*_min_ = 0.448, *T*_max_ = 0.692	*k* = −10→11
5003 measured reflections	*l* = −18→19
4088 independent reflections	3 standard reflections every 150 reflections
3359 reflections with *F*^2^ > 2.0σ(*F*^2^)	intensity decay: −0.6%

### Refinement

**Table:**

Refinement on *F*^2^	Secondary atom site location: difference Fourier map
*R*[*F*^2^ > 2σ(*F*^2^)] = 0.064	Hydrogen site location: inferred from neighbouring sites
*wR*(*F*^2^) = 0.204	H-atom parameters constrained
*S* = 1.07	*w* = 1/[σ^2^(*F*_o_^2^) + (0.1444*P*)^2^ + 3.2256*P*] where *P* = (*F*_o_^2^ + 2*F*_c_^2^)/3
4088 reflections	(Δ/σ)_max_ < 0.001
254 parameters	Δρ_max_ = 2.15 e Å^−^^3^
0 restraints	Δρ_min_ = −2.52 e Å^−^^3^
Primary atom site location: structure-invariant direct methods	

### Special details

**Table:**

Refinement. Refinement was performed using all reflections. The weighted *R*-factor (*wR*) and goodness of fit (*S*) are based on *F*^2^. *R*-factor (gt) are based on *F*. The threshold expression of *F*^2^ > 2.0 σ(*F*^2^) is used only for calculating *R*-factor (gt).

### Fractional atomic coordinates and isotropic or equivalent isotropic displacement parameters (Å^2^)

**Table:**

	*x*	*y*	*z*	*U*_iso_*/*U*_eq_	
Br1	0.50138 (8)	−0.13169 (6)	0.83532 (4)	0.0176 (2)	
Br2	0.31452 (8)	0.48290 (7)	0.92499 (4)	0.0201 (2)	
O1	0.4963 (6)	0.5714 (5)	0.7244 (4)	0.0187 (9)	
O2	0.7063 (7)	0.5130 (5)	0.5684 (3)	0.0202 (9)	
O3	0.6724 (7)	−0.0505 (6)	0.4014 (4)	0.0240 (10)	
N1	0.9928 (7)	0.1676 (6)	0.3998 (4)	0.0160 (10)	
N2	0.8285 (7)	0.1192 (6)	0.4157 (4)	0.0139 (9)	
C1	0.5910 (8)	0.3094 (6)	0.6818 (4)	0.0127 (10)	
C2	0.5037 (8)	0.4138 (7)	0.7463 (5)	0.0145 (11)	
C3	0.4201 (8)	0.3493 (7)	0.8375 (4)	0.0139 (11)	
C4	0.4218 (8)	0.1885 (7)	0.8626 (4)	0.0146 (11)	
C5	0.5058 (8)	0.0873 (7)	0.7989 (5)	0.0142 (11)	
C6	0.5922 (8)	0.1444 (7)	0.7097 (4)	0.0132 (10)	
C7	0.6900 (8)	0.3729 (7)	0.5888 (4)	0.0139 (10)	
C8	0.7757 (8)	0.2698 (7)	0.5188 (4)	0.0134 (10)	
C9	0.6977 (8)	0.1766 (7)	0.4881 (4)	0.0147 (11)	
C10	0.9595 (8)	0.2605 (7)	0.4605 (5)	0.0160 (11)	
C11	0.7991 (8)	0.0156 (7)	0.3655 (4)	0.0152 (11)	
C12	0.9184 (8)	0.0025 (7)	0.2726 (4)	0.0132 (10)	
C13	0.9601 (8)	0.1394 (7)	0.2138 (5)	0.0157 (11)	
C14	1.0545 (8)	0.1378 (7)	0.1201 (5)	0.0165 (11)	
C15	1.1092 (8)	−0.0030 (7)	0.0873 (4)	0.0135 (10)	
C16	1.1317 (8)	−0.2919 (8)	0.1108 (5)	0.0186 (12)	
C17	1.0949 (9)	−0.4298 (7)	0.1657 (5)	0.0187 (12)	
C18	0.9958 (9)	−0.4307 (7)	0.2580 (5)	0.0202 (12)	
C19	0.9346 (8)	−0.2926 (7)	0.2944 (5)	0.0161 (11)	
C20	1.0704 (8)	−0.1464 (7)	0.1456 (4)	0.0130 (10)	
C21	0.9702 (8)	−0.1460 (7)	0.2399 (4)	0.0135 (10)	
H1	0.3650	0.1466	0.9241	0.0175*	
H2	0.6521	0.0733	0.6672	0.0158*	
H3	0.5769	0.5890	0.6760	0.0225*	
H4	0.5756	0.1558	0.5125	0.0176*	
H5	1.0474	0.3148	0.4649	0.0192*	
H6	0.9250	0.2364	0.2367	0.0189*	
H7	1.0797	0.2336	0.0801	0.0198*	
H8	1.1739	−0.0043	0.0247	0.0162*	
H9	1.1988	−0.2924	0.0485	0.0223*	
H10	1.1363	−0.5261	0.1418	0.0224*	
H11	0.9707	−0.5279	0.2958	0.0243*	
H12	0.8676	−0.2959	0.3570	0.0193*	

### Atomic displacement parameters (Å^2^)

**Table:**

	*U*^11^	*U*^22^	*U*^33^	*U*^12^	*U*^13^	*U*^23^
Br1	0.0169 (4)	0.0074 (3)	0.0300 (4)	−0.0043 (2)	−0.0080 (3)	−0.0017 (3)
Br2	0.0234 (4)	0.0131 (4)	0.0230 (4)	−0.0001 (3)	−0.0030 (3)	−0.0092 (3)
O1	0.022 (2)	0.0063 (18)	0.031 (3)	−0.0053 (15)	−0.0045 (17)	−0.0060 (16)
O2	0.025 (3)	0.0088 (19)	0.027 (3)	−0.0068 (16)	−0.0039 (18)	−0.0027 (17)
O3	0.020 (2)	0.030 (3)	0.029 (3)	−0.0151 (19)	0.0060 (18)	−0.016 (2)
N1	0.013 (3)	0.016 (3)	0.022 (3)	−0.0056 (18)	−0.0070 (18)	−0.0047 (19)
N2	0.012 (2)	0.013 (3)	0.019 (3)	−0.0031 (17)	−0.0018 (18)	−0.0072 (18)
C1	0.010 (3)	0.007 (3)	0.023 (3)	0.0008 (18)	−0.007 (2)	−0.006 (2)
C2	0.013 (3)	0.007 (3)	0.028 (3)	−0.0048 (19)	−0.008 (2)	−0.004 (2)
C3	0.010 (3)	0.011 (3)	0.023 (3)	0.0010 (19)	−0.004 (2)	−0.010 (2)
C4	0.010 (3)	0.012 (3)	0.022 (3)	−0.000 (2)	−0.003 (2)	−0.008 (2)
C5	0.013 (3)	0.006 (3)	0.025 (3)	0.0006 (18)	−0.011 (2)	−0.002 (2)
C6	0.012 (3)	0.007 (3)	0.021 (3)	−0.0001 (18)	−0.007 (2)	−0.004 (2)
C7	0.013 (3)	0.010 (3)	0.018 (3)	−0.0016 (19)	−0.004 (2)	−0.004 (2)
C8	0.015 (3)	0.010 (3)	0.016 (3)	−0.0046 (19)	−0.004 (2)	−0.002 (2)
C9	0.017 (3)	0.010 (3)	0.019 (3)	−0.005 (2)	−0.004 (2)	−0.005 (2)
C10	0.017 (3)	0.009 (3)	0.023 (3)	−0.007 (2)	−0.006 (3)	0.000 (2)
C11	0.014 (3)	0.015 (3)	0.019 (3)	−0.003 (2)	−0.002 (2)	−0.009 (2)
C12	0.009 (3)	0.015 (3)	0.019 (3)	−0.0044 (19)	−0.0037 (19)	−0.005 (2)
C13	0.012 (3)	0.008 (3)	0.032 (4)	−0.0010 (19)	−0.012 (3)	−0.006 (3)
C14	0.016 (3)	0.011 (3)	0.023 (3)	−0.003 (2)	−0.009 (3)	−0.001 (2)
C15	0.010 (3)	0.015 (3)	0.017 (3)	−0.007 (2)	−0.0023 (19)	−0.002 (2)
C16	0.014 (3)	0.017 (3)	0.025 (3)	0.003 (2)	−0.005 (3)	−0.014 (3)
C17	0.019 (3)	0.009 (3)	0.031 (4)	0.001 (2)	−0.012 (3)	−0.008 (3)
C18	0.025 (3)	0.011 (3)	0.030 (4)	−0.008 (3)	−0.014 (3)	0.001 (3)
C19	0.017 (3)	0.013 (3)	0.020 (3)	−0.005 (2)	−0.008 (2)	−0.001 (2)
C20	0.011 (3)	0.011 (3)	0.018 (3)	−0.0032 (19)	−0.004 (2)	−0.004 (2)
C21	0.012 (3)	0.011 (3)	0.021 (3)	−0.0029 (19)	−0.008 (2)	−0.005 (2)

### Geometric parameters (Å, º)

**Table:**

Br1—C5	1.893 (6)	C13—C14	1.417 (9)
Br2—C3	1.870 (7)	C14—C15	1.373 (9)
O1—C2	1.341 (7)	C15—C20	1.417 (8)
O2—C7	1.243 (8)	C16—C17	1.356 (9)
O3—C11	1.213 (8)	C16—C20	1.425 (10)
N1—N2	1.368 (8)	C17—C18	1.404 (9)
N1—C10	1.317 (9)	C18—C19	1.378 (10)
N2—C9	1.364 (7)	C19—C21	1.416 (8)
N2—C11	1.427 (10)	C20—C21	1.434 (8)
C1—C2	1.419 (9)	O1—H3	0.840
C1—C6	1.417 (8)	C4—H1	0.950
C1—C7	1.475 (8)	C6—H2	0.950
C2—C3	1.415 (8)	C9—H4	0.950
C3—C4	1.379 (8)	C10—H5	0.950
C4—C5	1.389 (9)	C13—H6	0.950
C5—C6	1.380 (8)	C14—H7	0.950
C7—C8	1.473 (9)	C15—H8	0.950
C8—C9	1.367 (11)	C16—H9	0.950
C8—C10	1.426 (8)	C17—H10	0.950
C11—C12	1.476 (8)	C18—H11	0.950
C12—C13	1.382 (8)	C19—H12	0.950
C12—C21	1.438 (9)		
			
Br2···O1	3.001 (5)	H11···H12	2.3146
O1···O2	2.565 (7)	Br1···H7^iii^	3.3724
O1···C7	2.867 (8)	Br1···H8^viii^	3.4550
O2···C2	2.807 (7)	Br1···H10^ix^	3.3397
O2···C9	3.538 (9)	Br2···H6^v^	3.3350
O2···C10	2.996 (8)	Br2···H7^viii^	3.3550
O3···N1	3.512 (9)	Br2···H7^v^	3.1777
O3···C9	2.751 (10)	Br2···H9^x^	2.8779
O3···C13	3.512 (8)	Br2···H9^iii^	3.5074
O3···C19	2.944 (8)	Br2···H10^x^	3.1965
O3···C21	2.972 (7)	Br2···H10^ii^	3.5687
N1···C12	2.930 (10)	O1···H6^v^	3.0327
N1···C13	2.928 (10)	O1···H10^iii^	3.5909
N2···C13	2.926 (8)	O2···H4^v^	3.1289
C1···C4	2.795 (8)	O2···H5^vi^	2.6299
C1···C9	3.252 (9)	O2···H11^iii^	3.5241
C2···C5	2.803 (8)	O2···H12^iv^	3.3240
C3···C6	2.800 (9)	O2···H12^iii^	3.3782
C6···C8	2.958 (8)	O3···H2^ii^	2.8993
C6···C9	3.181 (9)	O3···H4^ii^	2.3008
C8···C11	3.570 (10)	O3···H5^iii^	3.1732
C10···C11	3.507 (11)	N1···H2^iii^	2.9620
C11···C19	2.994 (9)	N1···H11^iv^	2.7216
C12···C15	2.810 (8)	N2···H3^v^	3.5425
C13···C20	2.802 (9)	N2···H11^iv^	3.5371
C14···C21	2.848 (8)	C2···H11^ii^	3.4752
C16···C19	2.797 (9)	C3···H9^iii^	3.4520
C17···C21	2.827 (10)	C3···H11^ii^	3.5806
C18···C20	2.789 (9)	C4···H8^viii^	3.1593
Br1···O1^i^	3.492 (6)	C4···H8^iii^	3.4809
Br1···C12^ii^	3.542 (7)	C6···H12^ii^	3.4958
Br1···C14^iii^	3.477 (7)	C7···H12^iii^	3.3582
O1···Br1^iv^	3.492 (6)	C8···H11^iv^	3.5695
O1···N2^v^	3.564 (7)	C9···H3^v^	3.3636
O1···C17^iii^	3.481 (9)	C10···H11^iv^	2.6785
O1···C18^iii^	3.598 (8)	C10···H12^iii^	3.3923
O2···C9^v^	3.440 (7)	C11···H4^ii^	3.5045
O2···C10^vi^	3.558 (9)	C11···H5^iii^	3.4029
O2···C18^iii^	3.563 (10)	C12···H2^iii^	3.3072
O2···C19^iii^	3.476 (8)	C13···H2^iii^	3.4633
O3···O3^ii^	3.530 (7)	C13···H10^iv^	3.4537
O3···C6^ii^	3.253 (10)	C13···H11^iv^	3.5269
O3···C9^ii^	3.227 (8)	C14···H1^xi^	3.2807
O3···C10^iii^	3.520 (8)	C14···H9^vii^	3.2254
N1···N2^iii^	3.411 (7)	C14···H10^iv^	3.3528
N1···C9^iii^	3.455 (7)	C15···H1^xi^	3.0172
N2···O1^v^	3.564 (7)	C15···H8^vii^	2.9417
N2···N1^iii^	3.411 (7)	C15···H9^vii^	3.4108
N2···C10^iii^	3.451 (7)	C16···H1^ii^	3.5849
C1···C19^iii^	3.556 (9)	C16···H7^vii^	3.4040
C1···C21^iii^	3.432 (8)	C16···H8^vii^	3.4014
C2···C16^iii^	3.539 (10)	C17···H3^iii^	3.4810
C2···C17^iii^	3.566 (11)	C17···H6^i^	3.4220
C3···C16^iii^	3.421 (9)	C18···H3^iii^	3.2763
C3···C18^ii^	3.468 (10)	C18···H5^i^	3.3353
C4···C15^iii^	3.481 (8)	C18···H6^i^	3.2839
C5···C14^iii^	3.513 (8)	C19···H5^iii^	3.5826
C5···C15^iii^	3.422 (9)	C20···H8^vii^	3.2058
C5···C19^ii^	3.570 (9)	H1···C14^viii^	3.2807
C5···C21^ii^	3.544 (9)	H1···C15^viii^	3.0172
C6···O3^ii^	3.253 (10)	H1···C16^ii^	3.5849
C6···C12^iii^	3.470 (8)	H1···H1^xii^	3.3932
C6···C21^iii^	3.491 (10)	H1···H7^viii^	2.8501
C7···C19^iii^	3.395 (10)	H1···H8^viii^	2.2922
C9···O2^v^	3.440 (7)	H1···H8^iii^	3.4174
C9···O3^ii^	3.227 (8)	H2···O3^ii^	2.8993
C9···N1^iii^	3.455 (7)	H2···N1^iii^	2.9620
C10···O2^vi^	3.558 (9)	H2···C12^iii^	3.3072
C10···O3^iii^	3.520 (8)	H2···C13^iii^	3.4633
C10···N2^iii^	3.451 (7)	H3···N2^v^	3.5425
C10···C11^iii^	3.448 (8)	H3···C9^v^	3.3636
C10···C18^iv^	3.537 (8)	H3···C17^iii^	3.4810
C11···C10^iii^	3.448 (8)	H3···C18^iii^	3.2763
C12···Br1^ii^	3.542 (7)	H3···H4^v^	3.2835
C12···C6^iii^	3.470 (8)	H3···H5^vi^	3.2838
C14···Br1^iii^	3.477 (7)	H3···H11^iii^	3.3159
C14···C5^iii^	3.513 (8)	H4···O2^v^	3.1289
C15···C4^iii^	3.481 (8)	H4···O3^ii^	2.3008
C15···C5^iii^	3.422 (9)	H4···C11^ii^	3.5045
C15···C15^vii^	3.352 (10)	H4···H3^v^	3.2835
C16···C2^iii^	3.539 (10)	H4···H4^ii^	3.4472
C16···C3^iii^	3.421 (9)	H4···H12^ii^	3.4341
C17···O1^iii^	3.481 (9)	H5···O2^vi^	2.6299
C17···C2^iii^	3.566 (11)	H5···O3^iii^	3.1732
C18···O1^iii^	3.598 (8)	H5···C11^iii^	3.4029
C18···O2^iii^	3.563 (10)	H5···C18^iv^	3.3353
C18···C3^ii^	3.468 (10)	H5···C19^iii^	3.5826
C18···C10^i^	3.537 (8)	H5···H3^vi^	3.2838
C19···O2^iii^	3.476 (8)	H5···H5^vi^	3.5363
C19···C1^iii^	3.556 (9)	H5···H11^iv^	2.6349
C19···C5^ii^	3.570 (9)	H5···H12^iv^	3.3818
C19···C7^iii^	3.395 (10)	H5···H12^iii^	2.8249
C21···C1^iii^	3.432 (8)	H6···Br2^v^	3.3350
C21···C5^ii^	3.544 (9)	H6···O1^v^	3.0327
C21···C6^iii^	3.491 (10)	H6···C17^iv^	3.4220
Br1···H1	2.9064	H6···C18^iv^	3.2839
Br1···H2	2.9157	H6···H10^iv^	2.9117
Br2···H1	2.9029	H6···H11^iv^	2.6271
O2···H3	1.8478	H7···Br1^iii^	3.3724
O2···H5	2.9382	H7···Br2^xi^	3.3550
O3···H4	2.6228	H7···Br2^v^	3.1777
O3···H12	2.3649	H7···C16^vii^	3.4040
N1···H4	3.1758	H7···H1^xi^	2.8501
N1···H6	2.4781	H7···H9^vii^	2.9645
N2···H5	3.0438	H7···H10^iv^	2.7262
N2···H6	2.6233	H8···Br1^xi^	3.4550
C1···H3	2.4414	H8···C4^xi^	3.1593
C1···H4	3.2200	H8···C4^iii^	3.4809
C2···H1	3.2819	H8···C15^vii^	2.9417
C2···H2	3.3118	H8···C16^vii^	3.4014
C3···H3	3.0549	H8···C20^vii^	3.2058
C4···H2	3.2658	H8···H1^xi^	2.2922
C6···H1	3.2617	H8···H1^iii^	3.4174
C6···H4	2.9521	H8···H8^vii^	2.8061
C7···H2	2.6957	H8···H9^vii^	3.3014
C7···H3	2.4287	H9···Br2^xiii^	2.8779
C7···H4	2.9062	H9···Br2^iii^	3.5074
C7···H5	2.8364	H9···C3^iii^	3.4520
C8···H2	2.6214	H9···C14^vii^	3.2254
C9···H2	2.5715	H9···C15^vii^	3.4108
C9···H5	3.1225	H9···H7^vii^	2.9645
C10···H4	3.1375	H9···H8^vii^	3.3014
C10···H6	3.4812	H10···Br1^ix^	3.3397
C11···H4	2.7515	H10···Br2^xiii^	3.1965
C11···H6	2.6092	H10···Br2^ii^	3.5687
C11···H12	2.6897	H10···O1^iii^	3.5909
C12···H7	3.2902	H10···C13^i^	3.4537
C12···H12	2.7322	H10···C14^i^	3.3528
C13···H8	3.2701	H10···H6^i^	2.9117
C15···H6	3.2629	H10···H7^i^	2.7262
C15···H9	2.6357	H11···O2^iii^	3.5241
C16···H8	2.6301	H11···N1^i^	2.7216
C16···H11	3.2439	H11···N2^i^	3.5371
C17···H12	3.2752	H11···C2^ii^	3.4752
C18···H9	3.2522	H11···C3^ii^	3.5806
C19···H10	3.2731	H11···C8^i^	3.5695
C20···H7	3.2871	H11···C10^i^	2.6785
C20···H10	3.2758	H11···C13^i^	3.5269
C20···H12	3.3011	H11···H3^iii^	3.3159
C21···H6	3.3053	H11···H5^i^	2.6349
C21···H8	3.3269	H11···H6^i^	2.6271
C21···H9	3.3286	H12···O2^i^	3.3240
C21···H11	3.2836	H12···O2^iii^	3.3782
H2···H4	2.3745	H12···C6^ii^	3.4958
H6···H7	2.3618	H12···C7^iii^	3.3582
H7···H8	2.3192	H12···C10^iii^	3.3923
H8···H9	2.4494	H12···H4^ii^	3.4341
H9···H10	2.3002	H12···H5^i^	3.3818
H10···H11	2.3456	H12···H5^iii^	2.8249
			
N2—N1—C10	104.6 (5)	C14—C15—C20	121.0 (5)
N1—N2—C9	111.9 (6)	C17—C16—C20	120.6 (6)
N1—N2—C11	124.5 (5)	C16—C17—C18	120.2 (6)
C9—N2—C11	123.5 (6)	C17—C18—C19	121.0 (6)
C2—C1—C6	119.7 (5)	C18—C19—C21	120.8 (6)
C2—C1—C7	118.9 (5)	C15—C20—C16	120.0 (5)
C6—C1—C7	121.2 (5)	C15—C20—C21	120.3 (6)
O1—C2—C1	122.9 (5)	C16—C20—C21	119.7 (5)
O1—C2—C3	118.5 (6)	C12—C21—C19	125.0 (5)
C1—C2—C3	118.6 (5)	C12—C21—C20	117.3 (5)
Br2—C3—C2	119.1 (5)	C19—C21—C20	117.7 (6)
Br2—C3—C4	120.3 (5)	C2—O1—H3	109.471
C2—C3—C4	120.5 (6)	C3—C4—H1	119.719
C3—C4—C5	120.6 (5)	C5—C4—H1	119.718
Br1—C5—C4	119.3 (4)	C1—C6—H2	120.184
Br1—C5—C6	119.8 (5)	C5—C6—H2	120.182
C4—C5—C6	120.9 (6)	N2—C9—H4	126.528
C1—C6—C5	119.6 (6)	C8—C9—H4	126.542
O2—C7—C1	121.0 (6)	N1—C10—H5	124.031
O2—C7—C8	118.7 (5)	C8—C10—H5	124.022
C1—C7—C8	120.4 (5)	C12—C13—H6	119.423
C7—C8—C9	130.1 (5)	C14—C13—H6	119.416
C7—C8—C10	125.1 (7)	C13—C14—H7	120.217
C9—C8—C10	104.6 (6)	C15—C14—H7	120.214
N2—C9—C8	106.9 (6)	C14—C15—H8	119.513
N1—C10—C8	111.9 (6)	C20—C15—H8	119.515
O3—C11—N2	117.1 (6)	C17—C16—H9	119.699
O3—C11—C12	125.0 (7)	C20—C16—H9	119.707
N2—C11—C12	117.8 (6)	C16—C17—H10	119.889
C11—C12—C13	119.0 (6)	C18—C17—H10	119.880
C11—C12—C21	120.0 (5)	C17—C18—H11	119.506
C13—C12—C21	120.6 (5)	C19—C18—H11	119.498
C12—C13—C14	121.2 (6)	C18—C19—H12	119.584
C13—C14—C15	119.6 (6)	C21—C19—H12	119.586
			
H3—O1—C2—C1	−17.0	C9—C8—C10—H5	179.2
H3—O1—C2—C3	163.3	C10—C8—C9—N2	−0.7 (5)
N2—N1—C10—C8	1.9 (6)	C10—C8—C9—H4	179.3
N2—N1—C10—H5	−178.1	O3—C11—C12—C13	139.0 (6)
C10—N1—N2—C9	−2.4 (5)	O3—C11—C12—C21	−33.7 (8)
C10—N1—N2—C11	−179.9 (4)	N2—C11—C12—C13	−38.1 (7)
N1—N2—C9—C8	1.9 (6)	N2—C11—C12—C21	149.2 (5)
N1—N2—C9—H4	−178.1	C11—C12—C13—C14	−172.6 (5)
N1—N2—C11—O3	161.6 (5)	C11—C12—C13—H6	7.4
N1—N2—C11—C12	−21.1 (7)	C11—C12—C21—C19	−7.7 (9)
C9—N2—C11—O3	−15.7 (7)	C11—C12—C21—C20	174.2 (5)
C9—N2—C11—C12	161.7 (4)	C13—C12—C21—C19	179.8 (5)
C11—N2—C9—C8	179.5 (4)	C13—C12—C21—C20	1.7 (8)
C11—N2—C9—H4	−0.5	C21—C12—C13—C14	0.1 (9)
C2—C1—C6—C5	1.0 (9)	C21—C12—C13—H6	−179.9
C2—C1—C6—H2	−179.0	C12—C13—C14—C15	−1.4 (9)
C6—C1—C2—O1	−179.6 (5)	C12—C13—C14—H7	178.6
C6—C1—C2—C3	0.1 (9)	H6—C13—C14—C15	178.6
C2—C1—C7—O2	3.7 (9)	H6—C13—C14—H7	−1.4
C2—C1—C7—C8	−177.7 (5)	C13—C14—C15—C20	0.8 (9)
C7—C1—C2—O1	4.4 (9)	C13—C14—C15—H8	−179.2
C7—C1—C2—C3	−175.9 (5)	H7—C14—C15—C20	−179.2
C6—C1—C7—O2	−172.3 (5)	H7—C14—C15—H8	0.8
C6—C1—C7—C8	6.4 (9)	C14—C15—C20—C16	−178.9 (5)
C7—C1—C6—C5	176.9 (5)	C14—C15—C20—C21	1.0 (9)
C7—C1—C6—H2	−3.1	H8—C15—C20—C16	1.1
O1—C2—C3—Br2	−3.0 (8)	H8—C15—C20—C21	−179.0
O1—C2—C3—C4	179.0 (5)	C17—C16—C20—C15	−179.8 (6)
C1—C2—C3—Br2	177.3 (5)	C17—C16—C20—C21	0.4 (9)
C1—C2—C3—C4	−0.7 (9)	C20—C16—C17—C18	−0.0 (10)
Br2—C3—C4—C5	−177.8 (4)	C20—C16—C17—H10	180.0
Br2—C3—C4—H1	2.2	H9—C16—C17—C18	180.0
C2—C3—C4—C5	0.1 (9)	H9—C16—C17—H10	−0.0
C2—C3—C4—H1	−179.9	H9—C16—C20—C15	0.2
C3—C4—C5—Br1	−178.9 (5)	H9—C16—C20—C21	−179.6
C3—C4—C5—C6	1.1 (9)	C16—C17—C18—C19	−0.1 (10)
H1—C4—C5—Br1	1.1	C16—C17—C18—H11	179.9
H1—C4—C5—C6	−178.9	H10—C17—C18—C19	179.9
Br1—C5—C6—C1	178.4 (4)	H10—C17—C18—H11	−0.1
Br1—C5—C6—H2	−1.6	C17—C18—C19—C21	−0.1 (10)
C4—C5—C6—C1	−1.6 (9)	C17—C18—C19—H12	179.9
C4—C5—C6—H2	178.4	H11—C18—C19—C21	179.9
O2—C7—C8—C9	−132.1 (6)	H11—C18—C19—H12	−0.1
O2—C7—C8—C10	41.0 (8)	C18—C19—C21—C12	−177.6 (6)
C1—C7—C8—C9	49.2 (8)	C18—C19—C21—C20	0.5 (9)
C1—C7—C8—C10	−137.7 (5)	H12—C19—C21—C12	2.4
C7—C8—C9—N2	173.5 (5)	H12—C19—C21—C20	−179.5
C7—C8—C9—H4	−6.5	C15—C20—C21—C12	−2.2 (8)
C7—C8—C10—N1	−175.3 (5)	C15—C20—C21—C19	179.6 (5)
C7—C8—C10—H5	4.7	C16—C20—C21—C12	177.6 (5)
C9—C8—C10—N1	−0.8 (6)	C16—C20—C21—C19	−0.6 (8)

Symmetry codes: (i) *x*, *y*−1, *z*; (ii) −*x*+1, −*y*, −*z*+1; (iii) −*x*+2, −*y*, −*z*+1; (iv) *x*, *y*+1, *z*; (v) −*x*+1, −*y*+1, −*z*+1; (vi) −*x*+2, −*y*+1, −*z*+1; (vii) −*x*+2, −*y*, −*z*; (viii) *x*−1, *y*, *z*+1; (ix) −*x*+2, −*y*−1, −*z*+1; (x) *x*−1, *y*+1, *z*+1; (xi) *x*+1, *y*, *z*−1; (xii) −*x*+1, −*y*, −*z*+2; (xiii) *x*+1, *y*−1, *z*−1.

### Hydrogen-bond geometry (Å, º)

**Table:**

*D*—H···*A*	*D*—H	H···*A*	*D*···*A*	*D*—H···*A*
O1—H3···O2	0.84	1.85	2.565 (7)	142
C9—H4···O3^ii^	0.95	2.30	3.227 (8)	165
C16—H9···Br2^xiii^	0.95	2.88	3.613 (7)	135

Symmetry codes: (ii) −*x*+1, −*y*, −*z*+1; (xiii) *x*+1, *y*−1, *z*−1.
