# Supplementary figures and images for: Crystal structure of (3,5-dibromo-2-hy­droxyphenyl){1-[(naphthalen-1-yl)carbonyl]-1H-pyrazol-4-yl}methanone
Source: Acta Crystallogr Sect E Struct Rep Online. 2014 Aug 20;70(Pt 9):o1033. doi: 10.1107/S1600536814018601 (PMC4186182; doi:10.1107/S1600536814018601)

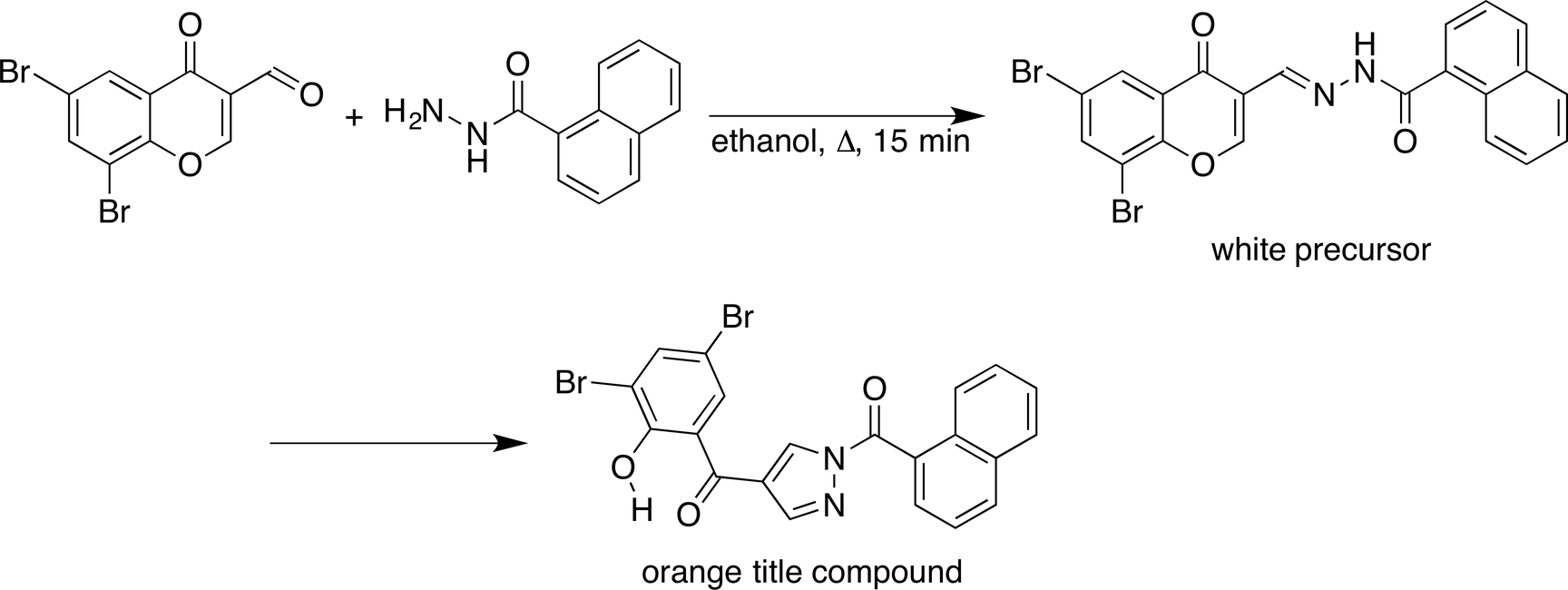

Supplement: Supplementary file 4 [file e-70-o1033-fig1.tif]

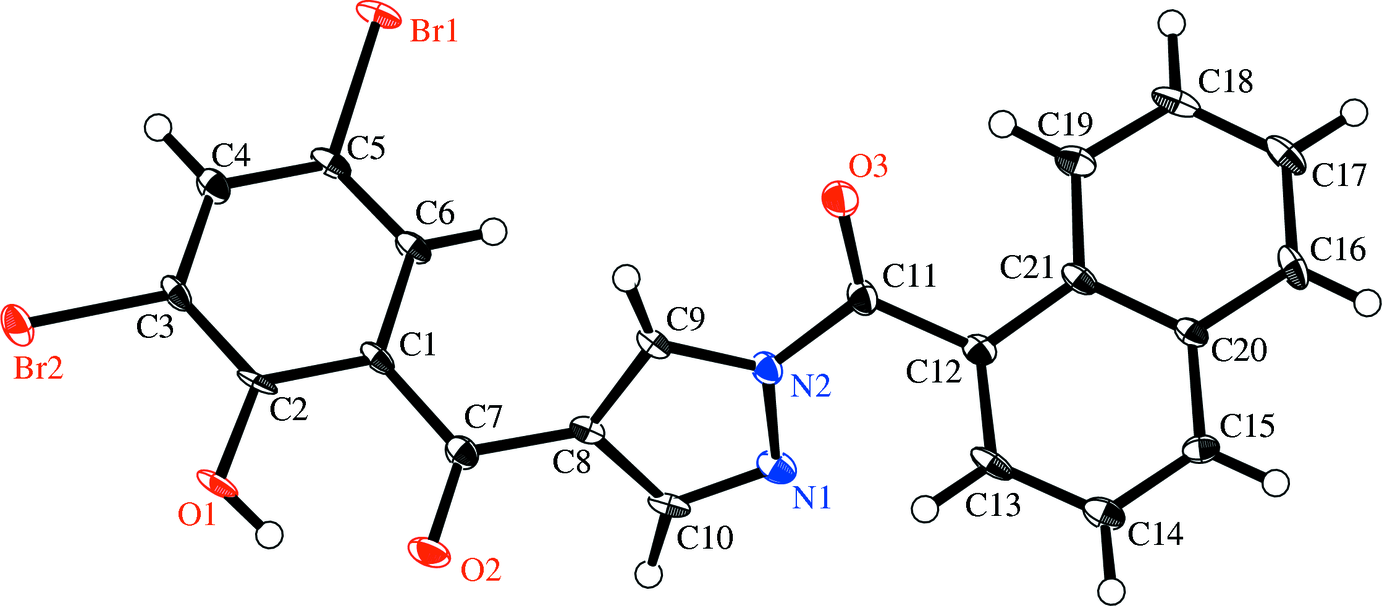

Supplement: Supplementary file 5 [file e-70-o1033-fig2.tif]

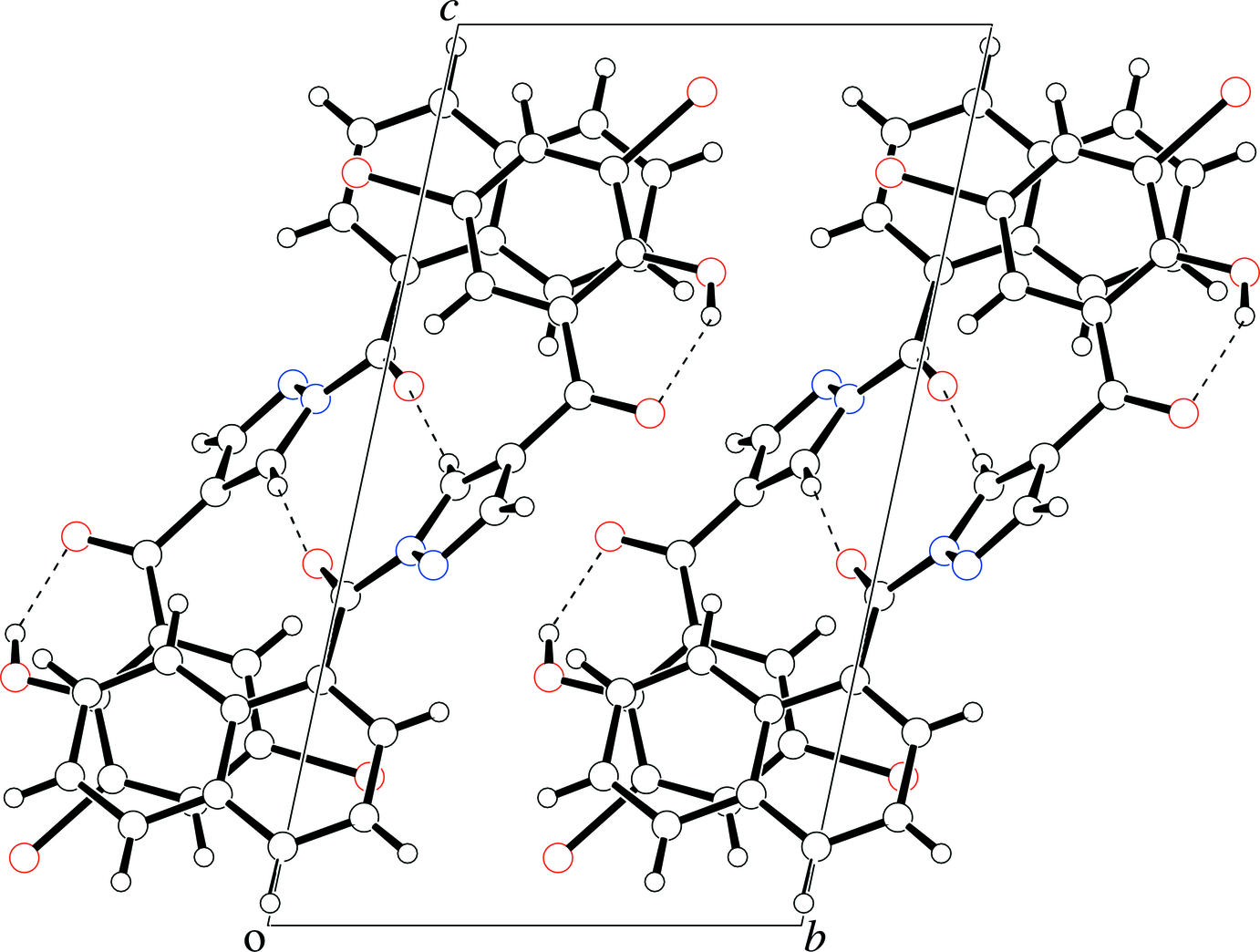

Supplement: Supplementary file 6 [file e-70-o1033-fig3.tif]
